# Insights into the diverse roles of the terminal oxidases in *Burkholderia cenocepacia* H111

**DOI:** 10.1038/s41598-025-86211-8

**Published:** 2025-01-18

**Authors:** Sarah Paszti, Olivier Biner, Yilei Liu, Kim Bolli, Sarah Dorothy Jeggli, Gabriella Pessi, Leo Eberl

**Affiliations:** Department of Plant and Microbial Biology, University of Zurich, Zollikerstrasse 107, Zürich, 8008 Switzerland

**Keywords:** Microbiology, Microbial genetics, Pathogens

## Abstract

*Burkholderia cenocepacia* H111 is an obligate aerobic bacterium which has been isolated from a cystic fibrosis (CF) patient. In CF lungs the environment is considered micro-oxic or even oxygen-depleted due to bacterial activities and limited oxygen diffusion in the mucus layer. To adapt to low oxygen concentrations, bacteria possess multiple terminal oxidases. In this study, we identified six terminal oxidases of *B. cenocepacia* H111 and constructed reporter strains to monitor their expression in different environments. While the heme-copper oxidase *aa*_*3*_ (*cta*) was constitutively expressed, the *bd*-1 oxidase (*cyd*) was induced under oxygen-limited growth conditions. The *c*yanide-*i*nsensitive *bd*-type terminal *o*xidase (*cio-1*) was mainly expressed in cells grown on the surface of solid medium or in liquid cultures in presence of cyanide, which is known to be produced in the CF lung by the often co-residing CF pathogen *Pseudomonas aeruginosa*. Indeed, a *cio-1* insertional mutant was not able to grow in the presence of cyanide confirming the important role of Cio-1 in cyanide resistance. The *caa*_*3*_ oxidase (*caa*), was only expressed under nutrient limitation when cells were grown on the surface of solid medium. We also investigated the involvement of two regulatory systems, Anr and RoxS/RoxR, in the expression of *cio-1* and *cyd*. Our data suggest, that, given that Cio-1 is only present in prokaryotes and plays an important role in the defense against cyanide-producing *P. aeruginosa*, it may be a valuable drug target for treatment of polymicrobial infections in CF patients.

**Supplementary Information:**

The online version contains supplementary material available at 10.1038/s41598-025-86211-8.

## Introduction

Cystic fibrosis (CF) patients are prone to chronic lung infections due to alterations of the mucus from smooth to thick and sticky^1–3^. The most important CF respiratory pathogen is *Pseudomonas aeruginosa*, which is linked to infections with high morbidity and mortality^4^. The environment of CF airways is heterogeneous in regard to oxygen levels, inflammation sites and nutrient availability. Furthermore, polymicrobial co-infections of *P. aeruginosa* and other pathogens such as *Staphylococcus aureus*, *Stenotrophomonas maltophilia*, *Streptococcus milleri* and members of the *Burkholderia cepacia complex* (*Bcc*) are common in CF patients^5^. Various oxygen concentrations have been reported in CF airways ranging from high (normoxic, aerobic) to low oxygen (microoxic, microaerobic) and even anoxic niches^6,7^. These oxygen gradients are further influenced by the consumption of oxygen by colonizing bacteria as well as host epithelial cells^3,8^. Many bacterial pathogens have adopted to these changing oxygen levels by incorporating several respiratory terminal oxidases with a range of oxygen affinities. For instance, *P. aeruginosa* encodes five different terminal oxidases^9,10^. Biochemically, terminal oxidases can be grouped into heme-copper oxidases and *bd*-type oxidases, depending on the redox cofactors recruited to catalyse the oxygen reduction^6,7^. Besides its different terminal oxidases, *P. aeruginosa* also uses different metabolic processes such as denitrification and fermentation of arginine and pyruvate to thrive in anoxic conditions^6^. *P. aeruginosa* is able to produce an arsenal of virulence factors (e.g., hydrogen cyanide (HCN), pyocyanin, elastase, rhamnolipids and other exotoxins) to shape microbial community compositions^11^. Interestingly, *P. aeruginosa* is one of the few bacteria known to be cyanogenic, meaning it can synthesize cyanide, which is a strong heme-copper oxidase inhibitor^8,12^. To circumvent intoxication of its own respiratory chain, *P. aeruginosa* possesses a cyanide-insensitive *bd*-type terminal oxidase (Cio)^13^. In soil-communities *P. aeruginosa* uses cyanide to outcompete other organisms and thereby protecting its niche^14^. It has been shown that the sputum of CF patients chronically infected with *P. aeruginosa* contains high concentrations of cyanide ranging from 72 µM to 130 µM^4,14^. Hydrogen cyanide synthesis by *P. aeruginosa* occurs under micro-aerobic conditions and is regulated by the CRP/FNR-type regulator Anr (anaerobic regulation of arginine deiminase and nitrate reduction) and by quorum sensing^15^.

In chronically infected CF patients, members of the *Bcc* are often co-isolated with *P. aeruginosa*^16^. Around 90% of *Bcc* infections in CF patients are caused by *Burkholderia cenocepacia* and *Burkholderia multivorans* strains^17^. Co-infections with members of the *Bcc* are associated with higher mortality sometimes related to the development of the “*cepacia* syndrome”, which is a systemic infection causing necrotizing pneumonia^18^. Infections with *Bcc* members are difficult to treat due to their intrinsic multi-resistance to antimicrobials. Proper treatment options to eradicate pulmonary infections with *Bcc* are only poorly developed^19^. For this reason, novel drug targets are urgently needed.

The clinical CF isolate *B. cenocepacia* H111^20^ is an obligate aerobe strictly relying on oxygen as final electron acceptor in its respiratory chain to grow. However, *B. cenocepacia* H111 can grow in microaerobic conditions with minimal oxygen concentrations of 0.1%^21^. In these conditions, expression of a cytochrome *bd*-type oxidase (*cyd*) is highly induced^21,22^. Whilst *B. cenocepacia* H111 is not able to grow without oxygen, it was shown that it can survive anoxia for several days^23^. A Tn-seq study, in which a mutant library was incubated anoxically for five days, identified 71 fitness determinants, which include transcriptional regulators such as Anr and the two-component regulatory system RoxS/RoxR as well as the cytochrome *bd*-type oxidase (Cyd)^23^. The transcriptional regulators Anr and RoxS/RoxR were previously shown to regulate the expression of the five terminal oxidase genes present in *P. aeruginosa*^24^.

Besides co-infection environments, interactions of *Bcc* members and *P. aeruginosa* are of importance in soil environments, but also in other environments such as premise plumbing or on medical devices^25,26^.

In contrast to *P. aeruginosa*, the terminal oxidases of *Bcc* members have so far not been investigated. In this study, we identified six terminal oxidases in *B. cenocepacia* H111 and constructed six reporter strains to study their expression patterns under different growth conditions. While the *bd*-1 oxidase (*cyd*) was important for growth of *B. cenocepacia* H111 under low oxygen, the cyanide insensitive oxidase 1 (*cio-1*) was found to be the main oxidase required for growth on agar plates as well as cyanide resistance. In line with this, a *cio-1* insertional mutant was unable to grow in the presence of cyanide, neither in vitro when supplementing the growth medium with cyanide nor in vivo in mixed culture with the cyanide-producing *P. aeruginosa* strain PA14. In addition, we showed that the two regulators Anr and RoxS/RoxR are key regulators of the expression of *cyd* and *cio-1* terminal oxidases.

## Results

### Identification of six terminal oxidase gene clusters in *B. cenocepacia* H111

**Fig. 1:**
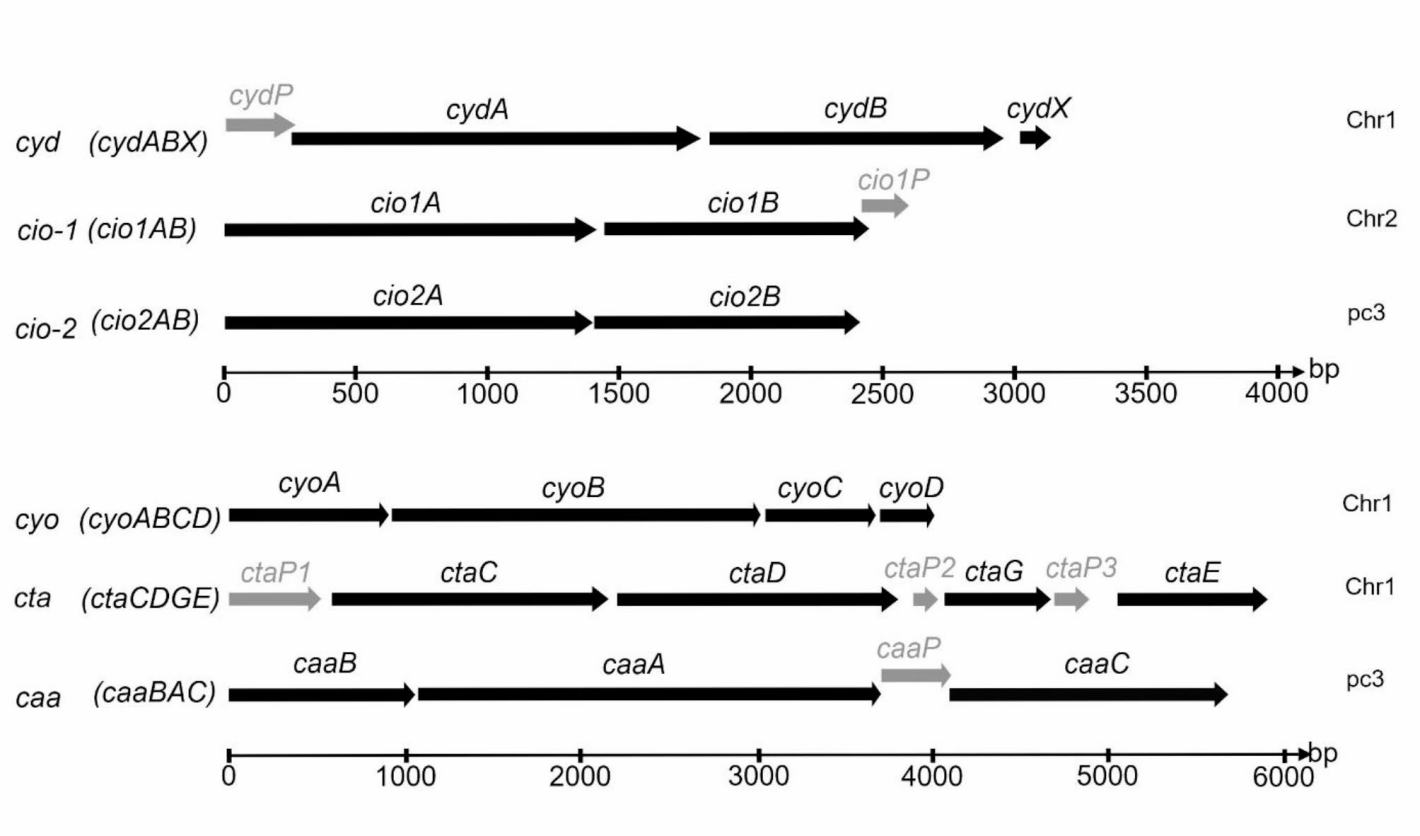
Gene cluster arrangement of the six identified terminal oxidases in *B. cenocepacia* H111. The chromosomal location is shown on the right. Grey marked loci are coding for previously not annotated potential small membrane proteins (SMPs): *bd-1* oxidase (*cyd* (*cydABX*), *I35_RS14435* to *I35_RS14445; cydA* shows a query cover of 86% and an Expect (E)-value of 1 × 10^−162^ compared to *E. coli* K-12 gene *cydA*), *cio-1* ((*cio1AB*), *I35_RS29260* and *I35_RS29265*) and *cio-2* ((*cio2AB*), *I35_RS33335* and *I35_RS33330*); *cio1A* and *cio2A* show query covers of 85% and 66% and E-values of 0.0 and 5 × 10^−145^, respectively, compared to *P. aeruginosa* PAO1 gene *cioA*), *bo*_*3*_ oxidase (*cyo* (*cyoABCD*), *I35_RS10365* to *I35_RS10350; cyoA* shows a query cover of 50% and an E-value of 9 × 10^−62^ compared to *P. aeruginosa* PAO1 gene *cyoA*), *aa*_*3*_ oxidase (*cta* (*ctaCDGE*), *I35_RS14585*, *I35_RS14580*,* I35_RS14570* and *I35_RS14560*) and *caa*_*3*_ oxidase (*caa* (*caaBAC*), *I35_RS32665* to *I35_RS32680*; *ctaD* and *caaA* show query covers of 71% and 38% and E-values of 6 × 10^−174^ and 9 × 10^−20^, respectively, compared to *P. denitrificans* PD1222 gene *ctaD)*.

The genome of *B. cenocepacia* H111 consists of two chromosomes and one mega plasmid (pc3) coding for approximately 6700 proteins^23,27^. The identification of gene clusters coding for terminal oxidases was conducted by taking known cytochrome oxidase encoding genes from different bacteria (e.g., *P. aeruginosa* PAO1, *Escherichia coli K-12*, *Paracoccus denitrificans* PD1222, or *Thermus thermophilus* HB8) and comparing them against the *B. cenocepacia* H111 genome (see Materials and Methods). This bioinformatic analysis revealed the presence of six loci coding for potential terminal oxidases (Fig. 1). Three loci code for heme-copper oxidases: a *bo*_3_ oxidase (*cyo*: *cyoABCD*), a *caa*_*3*_ oxidase (*caa*: *caaBAC*) and an *aa*_*3*_ oxidase (*cta*: *ctaCDGE*). The other three loci encode *bd*-type oxidases: a *bd*-1 oxidase (*cyd*: *cydABX*) and two *c*yanide-*i*nsensitive *o*xidases (*cio-1*: *cio1AB* and *cio-2*: *cio2AB*). While three of the terminal oxidases are located on chromosome 1 (*bo*_*3*_ (*cyo*), *aa*_*3*_ (*cta*) and *bd*-1 (*cyd*) oxidases), the *cio-1* oxidase is located on chromosome 2 and the *caa* and *cio-2* oxidases are located on pc3, which is known to be non-essential but confers stress resistance and is required for virulence^28,29^.

Interestingly, by closer inspection of the six operons coding for terminal oxidases, several open reading frames coding for potential small membrane proteins (SMPs), i.e., membrane proteins with ≤ 100 amino acids, were identified. SMPs are often not annotated in genomes because of their short open reading frame lengths. However, in the last decade it became apparent that SMPs are key regulators of cell membrane functioning, impacting the regulation and assembly of membrane proteins^30–32^. In *E. coli* K-12, the structure of *bd*-1 oxidase revealed, besides the two main subunits CydA and CydB, the presence of two additional SMP subunits: CydX and CydH, but only *cydX* is part of the *bd*-1 oxidase operon^33^. Similar to the *E. coli* K-12 *bd*-1 operon, the *B. cenocepacia* H111 *bd*-1 oxidase operon (*cyd*) contains genes coding for CydA, CydB and the CydX subunit (Table S1). In addition, a gene coding for a putative SMP subunit (*cydP*, *I35_RS14430*) was identified in the *bd*-1 operon of *B. cenocepacia* H111, preceding the *cydA* subunit. A similar gene encoding this subunit is absent in the *E. coli* K-12 *bd*-1 oxidase operon^23^. In contrast to *P. aeruginosa*, which has a single *cio* operon, *B. cenocepacia* H111 possesses two operons coding for cyanide-insensitive oxidases (*cio-1* and *cio-2*). The subunits Cio1A and Cio2A of *B. cenocepacia* H111 (92% coverage and 75% identity) as well as the subunits Cio1B and Cio2B (99% coverage and 58% identity)^34^ are highly similar, and therefore are considered as paralogs. Moreover, they are homologous to the cyanide-insensitive oxidase of *P. aeruginosa* PAO1 (Table S1). However, based on the operon structure, the Cio-1 terminal oxidase of *B. cenocepacia* H111 contains an additional putative SMP subunit, encoded by *cioP* (Fig. 1), that is not homologous to the *E. coli bd*-1 SMP *cydX* and is absent in the *cio* operon of *P. aeruginosa* and in *cio-2* of *B. cenocepacia* H111.

The *cyoABCD* cluster of *B. cenocepacia* H111 codes for a *bo*_3_ oxidase (*cyo*) and shares homology to the *cyoABCDE* cluster of *P. aeruginosa* PAO1 (Table S1). Unlike *P. aeruginosa*, the cluster of *B. cenocepacia* H111 does not possess a gene coding for subunit E (*cyoE*). No genes encoding potential SMPs were found in the *cyoABCD* cluster of *B. cenocepacia* H111. The three main subunits of the *B. cenocepacia* H111 *aa*_3_ oxidase (*cta*), CtaD, CtaC and CtaE are homologous to the *aa*_3_ oxidase of *P. denitrificans* PD1222 (Table S1). Additionally, we identified in *B. cenocepacia* H111 *ctaCDGE* operon a total of three SMPs subunits (CtaP1, CtaP2 and CtaP3), which are absent in *P. denitrificans* PD1222. The *caaBAC* operon of *B. cenocepacia* H111 codes for a *caa*_3_ oxidase (*caa*) and is homologous to the *caaAB* operon *of T. thermophilus* HB8 (Table S1). Both operons contain genes coding for the two main subunits CaaB and CaaA. Subunit CaaB is a fusion of a cytochrome *c* and the CuA containing subunit II (CtaC) while subunit CaaA is a fusion of the catalytic subunits I (CtaD) and subunit III (CtaE). In contrast to *T. thermophilus* HB8, the *caaA* gene of *B. cenocepacia* H111 is followed by a putative SMP encoding gene (*caaP*) and another gene (here labelled as *caaC*), annotated as a cytochrome *c*_533_, which are both absent in the operon of *T. thermophilus*.

### Expression of *cio-1* and *cyd* is induced in stationary phase and *cio-1* is the main terminal oxidase used when grown on solid agar plates

**Fig. 2:**
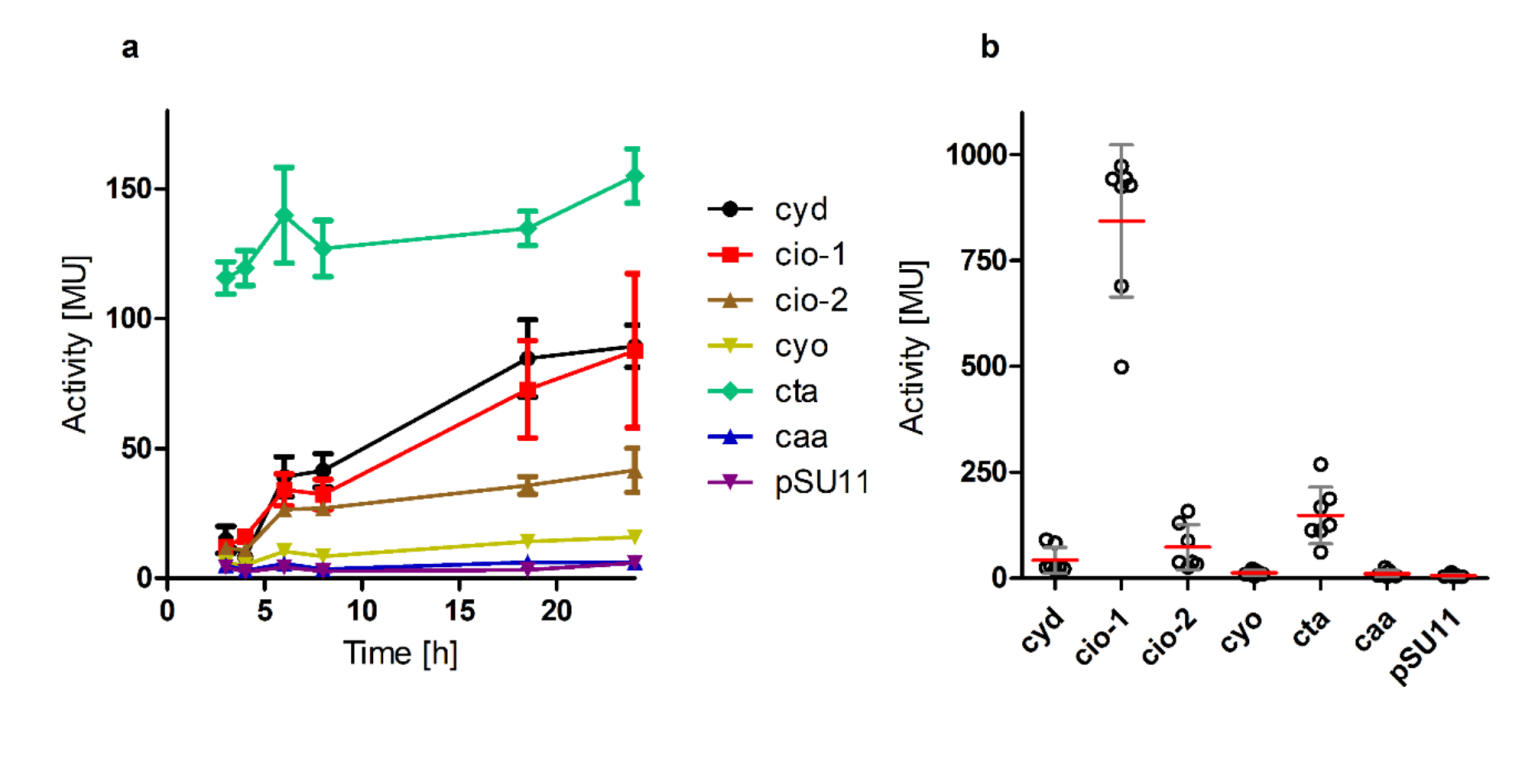
Expression patterns of the six terminal oxidases using *lacZ* reporter constructs. (**a**) Growth-dependent expression of the six terminal oxidases in liquid LB medium. (**b**) Expression pattern of the terminal oxidases grown on LB agar plates. MU = Miller units, Error bar = mean and standard deviation (SD) where *n* = 3 for (**a**) and *n* > 3 for (**b**). As a control the empty pSU11 plasmid was added into H111 (pSU11).

In this study, the promoters of the six terminal oxidases were fused with the *lacZ* reporter gene (see Materials and Methods) and used to assess the expression patterns of all six identified terminal oxidases in *B. cenocepacia* H111 in different growth conditions. As a first step, terminal oxidase expression was examined in aerobic growth condition in liquid cultures for 24 h (h) in either rich medium (LB) or minimal medium containing citrate as carbon source (ABC). In both media, the growth of the reporter strains was similar to the one of *B. cenocepacia* H111 wild-type strain containing the empty vector pSU11 (Fig. S1). In well aerated liquid LB medium cultures, the *aa*_3_ oxidase (*cta*) was expressed constitutively at high levels over the whole observed growth period (Fig. 2a and Table S2). Between the early exponential (3 h, OD_600_ ⌀ 0.4) and the late stationary phase (24 h, OD_600_ ⌀ 6.1) the expression of the *bd*-1 oxidase *cyd* and both cyanide-insensitive oxidases *cio-1* and *cio-2* were induced 6-, 7- and 3.5-fold, respectively (Fig. 2a and Table S2) and reached their highest level during late stationary phase, when the oxygen level and nutrient availabilities declined. In minimal medium, all reporter strains reached lower cell densities (Fig. S1). While the expression of the *bd*-1 oxidase (*cyd*) did not increase after 24 h of growth, *cio-1* and *cio-2* expression showed a less pronounced induction than in rich medium, (2- and 1.5-fold, respectively) (Fig. S2 and Table S3). The expression of the *bo*_3_ oxidase (*cyo*) and *caa*_3_ oxidase (*caa*) cluster was very low and not induced with increasing cell density, neither in rich nor in minimal medium (Fig. 2a and Fig. S2a). We showed by qPCR that *caa* is not expressed in cells grown in rich medium neither in exponential-nor in stationary phase (data not shown).

Next, the expression patterns of the terminal oxidases grown on plates on solid rich medium (LB) and minimal medium (ABC) was investigated. In nutrient rich growth conditions, *cio-1* was the predominantly expressed terminal oxidase with expression levels 70-fold and 10-fold higher compared to mid-exponential phase (3 h) and late stationary phase (24 h) growth in liquid LB medium, respectively. Moreover, *cio-1* expression on LB agar plates was 6-fold higher than the constitutively expressed *aa*_3_ oxidase (*cta*) (Fig. 2b). The expression of *cio-2* was only 2-fold higher when grown on solid agar than at late stationary phase (24 h) growth in liquid LB medium. We validated these results by qPCR in which we show that *cio-1* and *cio-2* expression is induced 4- and 8-fold, respectively, when cells were grown on solid agar compared to exponentially grown cells (Table S2). In ABC minimal medium, when grown on solid agar, *cio-1* expression was 29-fold higher than at mid-exponential phase (5 h) but reached lower overall levels than in rich medium (Fig. S2b and Table S3). The expression of the *caa* cluster in cells grown in minimal medium was 11- fold higher on plates compared to liquid medium (Fig. S2 and Table S3).

### The *bd*-1 oxidase (*cyd*) is expressed at low oxygen tensions

Terminal oxidases vary in their affinities for oxygen and thus are expressed in response to different oxygen tensions^35^. To test how the expression pattern of the terminal oxidases changes in regard to a reduction of the oxygen level in the medium, the oxygen-transfer coefficient of the medium was varied by changing the volume of the medium (LB) while keeping the flask volume (250 mL), shaking speed (200 rpm) and temperature (37 °C) constant as described by Cooper et al.^13^.

**Fig. 3:**
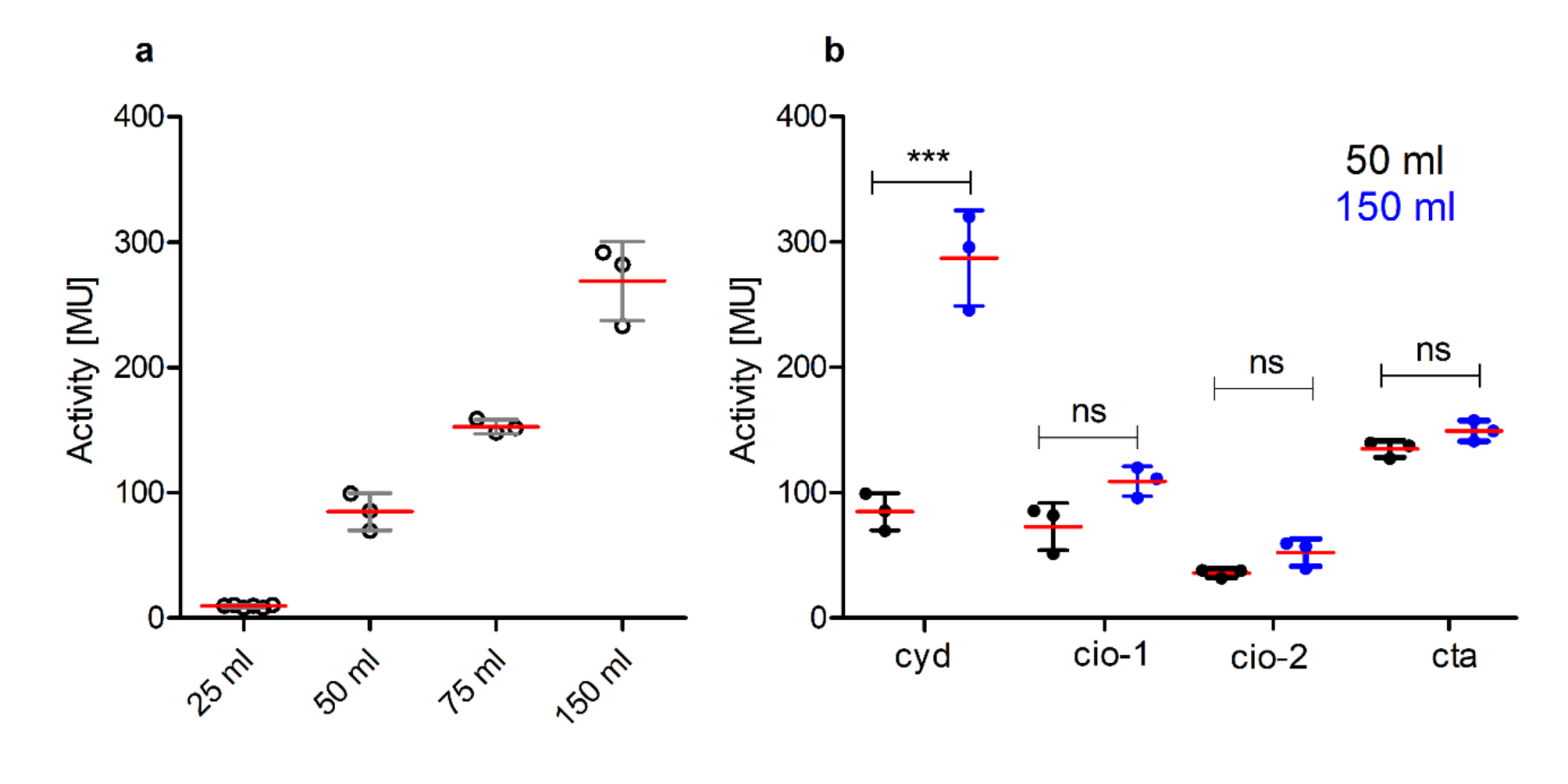
Expression of the terminal oxidases when *B. cenocepacia* H111 was grown at different oxygen concentrations. (**a**) *Bd-1* oxidase (*cyd)*-*lacZ* reporter strains were grown in 25 ml, 50 ml, 75 ml and 150 ml in 250 ml flasks for 20 h in LB medium at 200 rpm and at 37 °C. MU = Miller units, error bar = mean with SD, *n* = 3. (**b**) Expression pattern of the *bd-1* oxidase (*cyd*), *cio-1*,* cio-2* and the *aa*_*3*_ oxidase (*cta*) in 50 ml and 150 ml LB after 20 h shaken at 200 rpm at 37 °C. Statistical significance was calculated by one-way ANOVA using Tukey’s test, ***p < 0.001, ns = non-significant.

All six oxidase reporter strains were grown for 20 h in different media volumes (25 ml, 50 ml, 75 ml and 150 ml) in a 250 ml flask. The expression of the *bd*-1 oxidase (*cyd*) was linearly dependent on the media volume reaching a 30-fold induction in the culture containing reduced oxygen (150 ml) compared to the fully aerobically grown culture (25 ml) (Fig. 3a). Previous RNA-seq data from our group identified *cyd* as one of the *B. cenocepacia* H111 genes with induced expression in a low-oxygen environment^21^. In contrast, the expression of the *aa*_3_ oxidase (*cta*), *cio-1* and *cio-2* did not change significantly when cells were grown at different oxygen tensions (Fig. 3b and Table S4). The *bo*_3_ oxidase (*cyo*) and *caa*_3_ oxidase (*caa*) were not expressed at any of the tested oxygen concentrations (Table S4).

### *B. cenocepacia* H111 Cio-1 confers resistance to cyanide

**Fig. 4:**
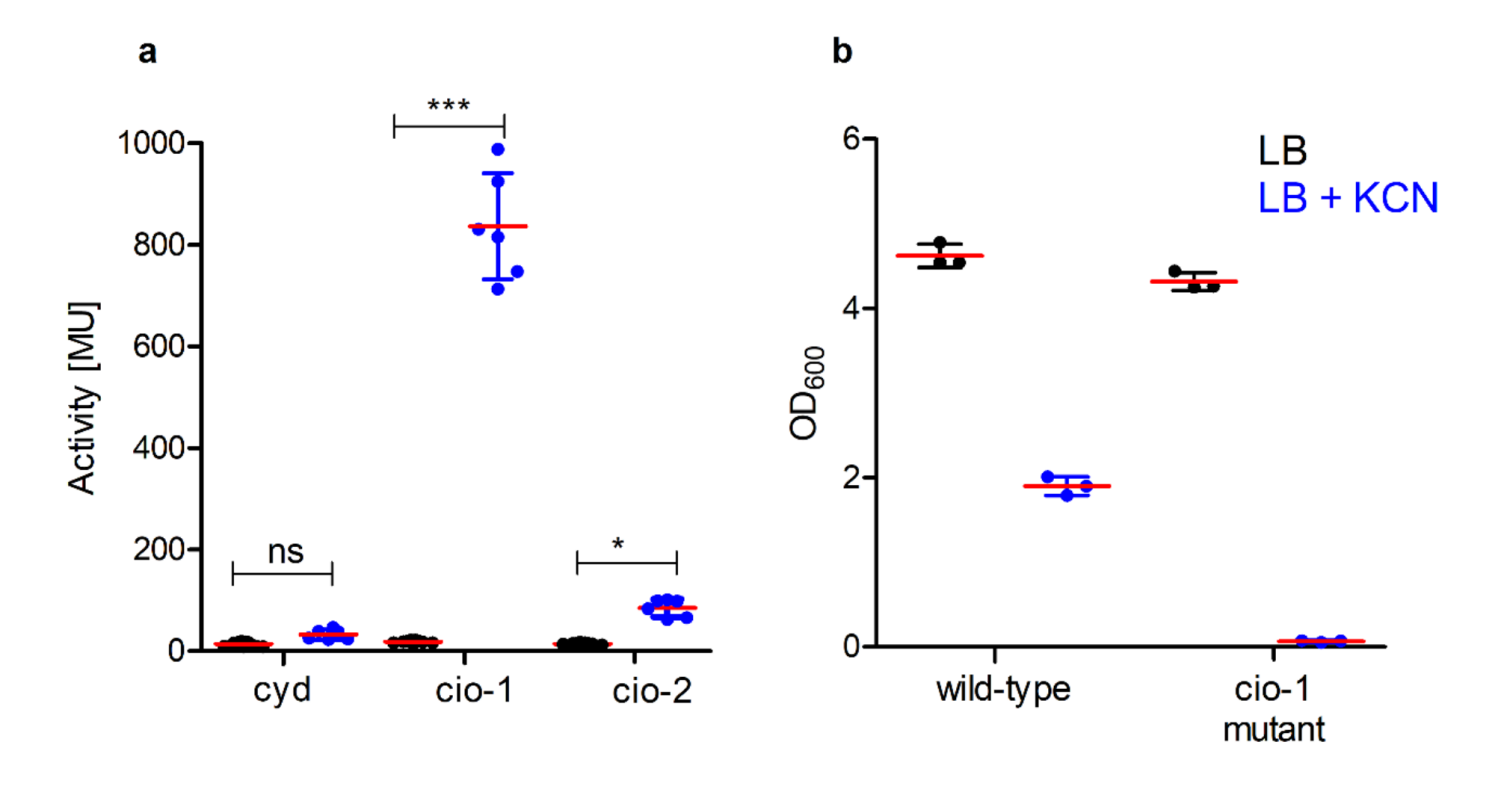
Effect of cyanide on terminal oxidase expression and growth of *B. cenocepacia* H111 wild-type and the *cio-1* mutant strain. (**a**) Expression pattern of the *bd-1* oxidase (*cyd*), *cio-1*, and *cio-2* in LB and LB amended with 600 µM KCN at OD_600_ 0.3–0.5. MU = Miller units, mean with SD, *n* > 3. Statistical significance was calculated by one-way ANOVA using Tukey’s test, ***p < 0.001, ns = non-significant. (**b**) Growth (OD_600_) of wild-type and *cio-1* insertional mutant in LB and LB supplemented with 600 µM KCN after 24 h.

*B. cenocepacia* H111 cyanide-insensitive oxidase *cio-1* was the most expressed terminal oxidase on plate (Fig. 2b). To investigate if - similarly to the situation in *P. aeruginosa*- *B. cenocepacia* H111 *cio-1* was involved in cyanide resistance, 600 µM potassium cyanide (KCN) were added to liquid LB medium and the expression of the different terminal oxidase reporter strains was measured. Indeed, *cio-1* expression was induced around 40-fold when *B. cenocepacia* H111 was grown in presence of exogenous KCN (Fig. 4a and Table S4). The cluster coding for the second cyanide-insensitive oxidase *cio-2* was 6-fold up-regulated by cyanide addition, while no change in expression was observed for the *bd*-1 oxidase (*cyd).* These results were validated by qPCR analysis, which showed that *cio-1* and *cio-2* expression were induced 66- and 43 fold in the presence of KCN compared to exponentially grown cultures (Table S6).

**Fig. 5:**
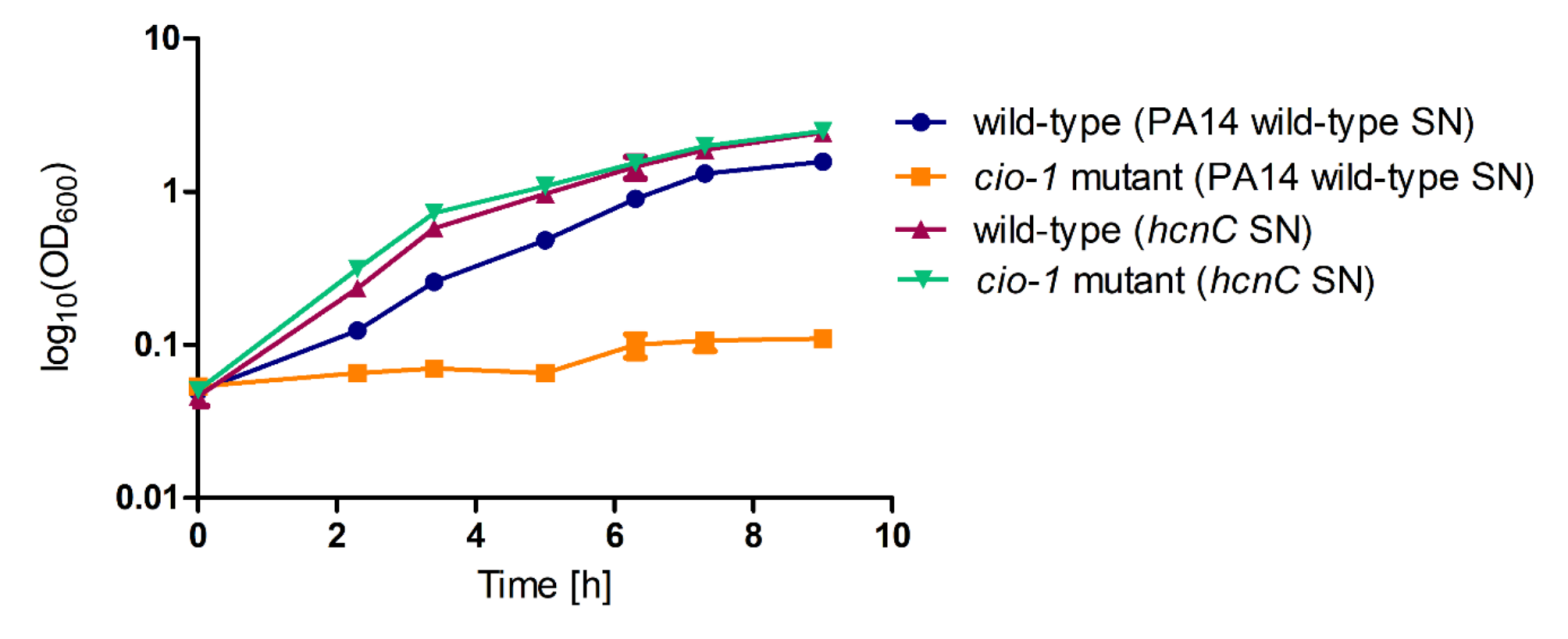
Supernatant (SN) assay with *B. cenocepacia* H111 wild-type and the *cio-1* insertional mutant. Supernatant of *P. aeruginosa* PA14 wild-type (cyanide producer) and *P. aeruginosa* PA14 *hcnC* mutant (cyanide negative) mixed with 50% fresh LB medium were tested. X-axis time in h and y-axis log_10_(OD_600_). Error bars mean with SD, *n* = 3.

To test the role of *cio-1* in cyanide resistance, a *cio-1* insertional mutant was created and tested for growth in presence of KCN (600 µM). The wild-type strain showed impaired growth in the presence of exogenous KCN reaching an average OD_600_ of 1.9 after 24 h compared to an OD_600_ of 4.62 when grown without cyanide addition. The *cio-1* insertional mutant grew as well as the wild-type but was not able to grow in a medium containing exogenous KCN (Fig. 4b). To test if *cio-1* may give *B. cenocepacia* H111 a competitive advantage by conferring resistance to cyanide produced by *P. aeruginosa*, the supernatant from *P. aeruginosa* PA14 wild-type strain and a *P. aeruginosa* PA14 *hcnC* mutant strain grown in liquid LB medium overnight were collected. The supernatant of *P. aeruginosa* PA14 wild-type or *hcnC* mutant was then mixed with fresh LB medium at a 1:1 ratio and inoculated either with *B. cenocepacia* H111 or the *cio-1* insertional mutant strain (Fig. 5)^36^. While *B. cenocepacia* H111 wild-type strain was able to grow in the presence of the supernatant from *P. aeruginosa* PA14 wild-type and *hcnC* mutant, the *B. cenocepacia* H111 *cio-1* insertional mutant grew only in presence of the supernatant of the *P. aeruginosa hcnC* mutant strain, which did not contain cyanide. This result is consistent with the observation that exogenous KCN restricts the growth of the *cio-1* insertional mutant strain.

### The two-component system RoxS/RoxR and the transcriptional regulator Anr are involved in the regulation of *cyd* and *cio-1* in *B. cenocepacia* H111

To test the involvement of the transcriptional regulator Anr and the two-component regulatory system RoxS/RoxR in controlling the expression of the terminal oxidases of *B. cenocepacia* H111, the reporter plasmids were conjugated into previously constructed deletion mutant strains^23^. Since *B. cenocepacia* H111 possesses two *anr* genes (*anr*_*1*_: *I35_RS16810* and *anr*_*2*_: *I35_RS23120*), we used a ∆*anr*_*1*_*-anr*_*2*_ double knockout mutant. For the two-component system RoxS/RoxR, the sensor kinase mutant ∆*roxS* strain was used^23^. The expression of each terminal oxidase in *B. cenocepacia* H111 wild-type, ∆*anr*_*1*_*-anr*_*2*_ and ∆*roxS* was assessed in LB liquid culture and on the surface of an LB agar plate. While Anr repressed the expression of *cio-1*, the expression of the *bd*-1 oxidase (*cyd*) was positively controlled by Anr in both growth conditions (Table 1). In the absence of RoxS the expression of *cyd* and *cio-1* decreased indicating that two-component system RoxS/RoxR positively regulates these two terminal oxidases. The expression of the other terminal oxidases was not affected by *anr* or *roxS* deletions (Table S5).

**Table 1:** RoxS/RoxR and anr-dependent expression of the six terminal oxidases in *B. cenocepacia* H111. The *B. cenocepacia* H111 reporter strains were grown in LB liquid and solid LB medium. For liquid culture *n* ≥ 3 and for cells grown on LB agar plates *n* ≥ 5. The values are given in Miller Units.

Terminal oxidase	Liquid culture			Plate		
	Wild-type	∆*roxS*	∆*anr*_*1*_*anr*_*2*_	Wild-type	∆*roxS*	∆*anr*_*1*_*anr*_*2*_
*cyd*	82 ± 25	6 ± 1	1 ± 0	42 ± 28	5 ± 1	4 ± 2
*cio*-1	43 ± 11	9 ± 0	1358 ± 557	843 ± 166	9 ± 4	1498 ± 281
*cio-2*	57 ± 11	53 ± 3	42 ± 4	73 ± 49	32 ± 6	39 ± 8
*cyo*	17 ± 8	7 ± 1	24 ± 3	12 ± 5	4 ± 2	9 ± 4
*cta*	228 ± 53	207 ± 31	145 ± 7	148 ± 62	103 ± 15	125 ± 31
*caa*	3 ± 3	3 ± 1	4 ± 2	11 ± 7	4 ± 2	4 ± 3
Control (pSU11)	3 ± 2	2 ± 1	1 ± 0	6 ± 3	3 ± 2	2 ± 1

**Fig. 6:**
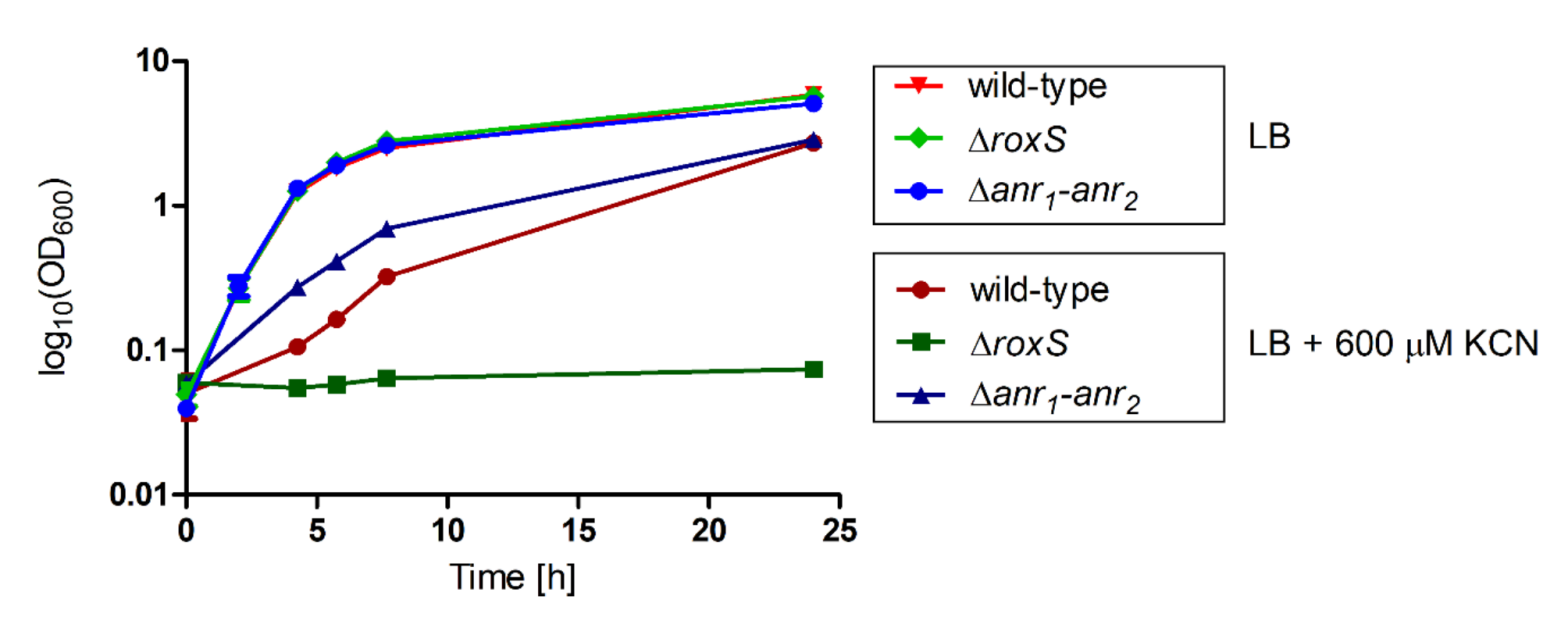
Growth of *B. cenocepacia* H111 wild-type and Δ*roxS* and Δ*anr*_*1*_*anr*_*2*_ in the presence of cyanide. Aerobic growth of the wild-type, ∆*anr*_*1*_*-anr*_*2*_ and ∆*roxS* mutants in LB and LB supplemented with 600 µM KCN for 24 h at 200 rpm at 37 °C. Error bars mean with SD, *n* = 3.

Next, the growth of the ∆*anr*_*1*_*-anr*_*2*_ and ∆*roxS* mutants was measured in LB and LB amended with 600 µM KCN and compared to the wild-type strain (Fig. 6). In LB all strains showed similar growth. In presence of cyanide, the ∆*anr*_*1*_*-anr*_*2*_ deletion mutant strain grew faster than the wild-type for the first 8 h but reached similar densities after 24 h of growth. In contrast, the ∆*roxS* mutant was not growing in the presence of cyanide, confirming the importance of this two-component regulatory system in activating expression of *cio-1*.

## Discussion

**Fig. 7:**
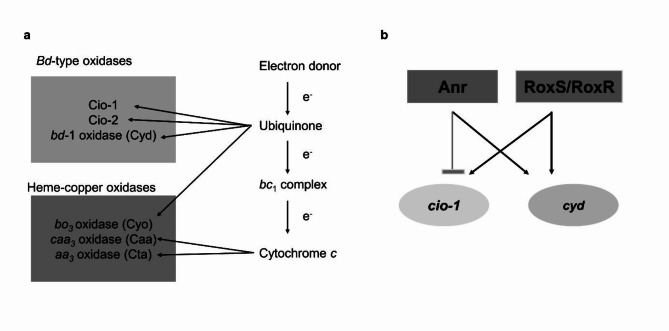
Graphics showing the terminal oxidases of *B. cenocepacia* H111. (**a**) Overview of the terminal oxidases of *B. cenocepacia* H111 and their electron donors. (**b**) Graph showing the involvement of Anr and RoxS/RoxR in the regulation of *cio-1* and *cyd* terminal oxidases in *B. cenocepacia* H111. Black arrows show positive regulation and grey line shows negative regulation.

In this study we identified a total of six operons coding for terminal oxidases in the genome of the opportunistic CF pathogen *B. cenocepacia* H111 and out of these six, three were heme-copper oxidases (*cta*, *caa*, *cyo*) and three *bd*-type oxidases (*cyd*,* cio-1*,* cio-2*) (Fig. 7a).

The main terminal oxidase of *B. cenocepacia* H111 appears to be the *aa*_3_ oxidase (*cta*), which was constitutively expressed at high levels throughout all tested conditions. The *aa*_3_ oxidases are heme-copper oxidases that use cytochrome *c* as electron donor and typically have low oxygen affinities but high proton translocation stoichiometries enabling efficient energy transduction^9,37,38^. By contrast, the *caa*_3_ (*caa*) and *bo*_3_ (*cyo*) oxidases were not expressed in rich medium neither in both liquid culture nor on agar plates. In *P. aeruginosa*, the *caa*_3_ oxidase (*caa*) was expressed during nutrient starvation in glucose synthetic medium and the *bo*_3_ oxidase under iron starvation^39^. However, we did not see expression of either oxidase when *B. cenocepacia* H111 was grown in liquid minimal media with limiting carbon sources (citrate or glucose) or under iron depletion (data not shown). Furthermore, we tested if a decrease in pH leads to an induction of *cyo* expression, as *cyo* in *Rhizobium etli* CFN42 is induced at low pH^40^. However, shifting the pH from 7.0 to 5.0 did not induce *cyo* expression (data not shown). An 11-fold induction of expression of *caa* was observed when *B. cenocepacia* was grown on agar plates with ABC minimal medium, indicating that the *caa*_3_ oxidase might be important for growth in a biofilm. In this study, we confirm that the expression of the *cyd* operon is 30-times induced when *B. cenocepacia* H111 was grown under reduced oxygen tension compared to fully aerated cells. Cytochrome *bd* oxidases are known to have higher oxygen affinities than heme-copper oxidases and are used for growth under low oxygen conditions^41–43^. The increased expression of the *cyd* operon in the late stationary phase can probably be attributed to the reduced oxygen availability in this growth phase. Besides *cyd*, *B. cenocepacia* H111 possesses two other *bd*-type terminal oxidases, *cio-1* and *cio-2*, which both were up-regulated in stationary phase but were not induced under low oxygen conditions. In contrast to *bd*-1 oxidases that have low *K*_m_ values for oxygen binding (range of 3–20 nM^9,44,45^), the *K*_m_ values of known *Cio’s* are 20-100× fold higher: 0.41 µM for *P. aeruginosa*^9^, 0.8 µM for *Campylobacter jejuni*^46^ and 20 µM for *Gluconobacter oxydans*^47^. We hypothesize that *cyd* may be the main terminal oxidase used by *B. cenocepacia* H111 under low oxygen tensions. The expression of *cio*-*1* and *cio-2* significantly increased after addition of cyanide, however, the induction was much stronger for *cio-1* than for *cio-2* despite the close sequence similarity of these operons. In line with these results, the *cio-1* insertion mutant strain lost its ability to grow in the presence of cyanide. Cyanide is a potent inhibitor of heme-copper oxidases, with IC_50_ values typically in the low micromolar range^48,49^. Cio-1 may allow *B. cenocepacia* H111 to produce a proton motive force in the presence of high cyanide concentrations, which have been reported to occur in the lungs of CF patients infected with *P. aeruginosa*^4,8,50^. Hence, Cio-1 might be important for establishing mixed infections. In support of this hypothesis, we showed that the *cio-1* insertion mutant did not grow in presence of *P. aeruginosa* PA14 supernatant, whereas the *B. cenocepacia* H111 wild-type strain did. This growth inhibitory effect was not observed when the *cio-1* mutant was incubated with the supernatant of a *P. aeruginosa* PA14 *hcnC* mutant, which does not produce cyanide. Collectively, our data suggest that cyanide resistance in *B. cenocepacia* H111 conferred by *cio-1* may be important for forming mixed biofilms of *B. cenocepacia* and *P. aeruginosa* in the CF lung and in others shared environments such as human-influenced soil or premise plumbing^51–53^. Direct co-culturing studies will be an important future direction to gain further insight into the physiological roles of terminal oxidases in mixed biofilms.

Interestingly, we also observed induction of *cio*-*1* and *cio-2* expression in the absence of cyanide at the entry of the stationary phase and on plates. One possible explanation for this observation is that *B. cenocepacia* H111 also produces cyanide under these conditions. In fact, in *P. aeruginosa*, cyanide production is activated by quorum sensing and low oxygen levels^50^. Cyanide production has been reported for some members of the *Bcc* including *B. cenocepacia* J2315 under biofilm growth conditions^54^. Three homologues of the *P. aeruginosa* cyanide synthase (*hcn*) genes were identified in the *B. cenocepacia* J2315 genome^54^. However, we and other groups were not able to detect cyanide when *B. cenocepacia* H111 was grown in rich medium to stationary phase or on agar plates^55,56^. *P. aeruginosa* has been proposed to produce cyanide at high cell densities as a policing agent to restrain cheaters and to protect its ecological niche by inhibiting respiration in competing organisms. In fact the *cio* operon has been shown to provide selective protection to cooperators^57^. Furthermore, *P. aeruginosa* was shown to control growth of *S. aureus* in a mouse lung infection model by producing cyanide, which plays a key role in shaping the polymicrobial composition in CF lungs^58^. *S. aureus* is known to be an important colonizer of the CF lung in early stages of the disease and is later often replaced by *P. aeruginosa*. A previous transcriptome analysis of the two *B. cenocepacia* CF isolates, *B. cenocepacia* IST439 (started the initial infection) and the clonal variant *B. cenocepacia* IST4113 (three years after initial infection representative of a chronic infection) showed that in the late isolate *B. cenocepacia* IST4113 *cio-1* was upregulated^59^. Likewise, a Tn-seq study showed that in *B. pseudomallei cio-1* but not *cio-2* is important for lung colonization^60^.

The expression of terminal oxidases is tightly regulated to allow adaptation to specific niches. In *P. aeruginosa* the oxygen sensor Anr and the two-component system RoxS/RoxR, are known to coordinate the expression of its five terminal oxidases^24^. It is worth mentioning that *B. cenocepacia* has two copies of Anr, Anr_1_ and Anr_2_^23^. We did not discriminate between those in this study and all experiments were performed in a double knock-out mutant (∆*anr*_*1*_*-anr*_*2*_). We found that both *cyd* and *cio-1* are regulated by Anr and RoxS/RoxR (Fig. 7b). The expression of the *bd*-1 oxidase (*cyd*) was positively regulated by Anr and RoxS/RoxR. This is in agreement with the finding that the high oxygen affinity *cbb*_3_-2 oxidase of *P. aeruginosa* is also positively regulated by these two regulatory systems. Similarly, to the situation in *P. aeruginosa*, the expression of the *cio-1* operon was positively regulated by RoxS/RoxR but inhibited by Anr. Cio’s are known to have low oxygen affinities^9,46,47^ and are thus not well suited for low oxygen conditions^23,61^. However, Anr positively regulates expression of *cyd*, the high oxygen affinity terminal oxidase in *B. cenocepacia* H111. RoxS/RoxR is known to respond to the overall redox state of the respiratory chain^62^. We found that deletion of *roxS* resulted in a loss of cyanide sensitivity, probably due to the positive regulation of *cio-1* by RoxS/RoxR. In the presence of cyanide, the redox state of the electron transfer chain changes. Instead of electron transfer from the ubiquinone pool to the *bc*-1 complex, cytochrome *c* and the *aa*_3_-oxidase, the reduced ubiquinone can directly be re-oxidized by a ubiquinol terminal oxidase (such as *cio-1* or *cyd*). Thus, it can be hypothesized that RoxS/RoxR senses the redox state of the ubiquinone pool. A positive regulation of *cio-1* and *cyd* by the Rox system is in line with this hypothesis.

Bacterial energy production is an emerging target for the development of antimicrobial drugs^63^. For example, a terminal oxidase inhibitor drug (Telacebec (Q203)) in combination with *bd*-type oxidase inhibitors were recently shown to effectively eradicate multidrug-resistant *M. tuberculosis* infections^64^. The fact that *bd*-type oxidases are only present in prokaryotes make them highly attractive drug targets^65^. Our study characterized the terminal oxidases of *B. cenocepacia*, which may pave the way for the future development of inhibitors for treatment of *B. cenocepacia* infections.

## Materials and methods

### Bioinformatic analysis

Genes of known terminal oxidases were compared to the refseq_genomes database limited to entries of *B. cenocepacia* H111 (taxid: 1055524) using different BLAST searches^66–68^. The *E. coli* K-12 gene *cydA* (NCBI reference sequence: NC_000913.3: 771458.773026), the *P. aeruginosa* PAO1 genes *cioA* (NC_002516.2: 4404902.4406368, complement) and *cyoA* (NC_002516.2: 1428080.1429075) were compared using (discontinuous) megablast searches. The *P. denitrificans* PD1222 gene *ctaD* (NC_008686.1: 1937038.1938714) was compared using a blastn search.

### Bacterial strains, media, and growth conditions

All bacterial strains, oligonucleotides and primers are listed in Table S7. If not stated otherwise, bacterial strains were grown aerobically at 37 °C either by shaking at 200 rpm or stationary in rich medium LB^69^ or minimal medium AB^70^ supplemented with 10 mM sodium citrate (ABC) as a carbon source. If required for selection, bacterial cultures were supplemented with antibiotics at the following concentrations: Gentamycin (Gm) 10 µg/mL, chloramphenicol (Cm) 80 µg/mL, kanamycin (Kn) 50 µg/mL.

### Construction of *lacZ* reporter strains

Transcriptional fusions of the promoters of the terminal oxidase genes with *lacZ* were constructed using the plasmid pSU11 carrying the *lacZ* gene^71,72^. Shortly, the promoters of the terminal oxidase operons were amplified by PCR using the listed primer pairs (Table S7) and the isolated genomic DNA of *B. cenocepacia* H111 as template. The genomic DNA of *B. cenocepacia* H111 was extracted using the GenElute™ Bacterial Genomic DNA Kit (Sigma-Aldrich, NA2110). The amplified fragments (fragment lengths can be found in Table S7) were cut using prior introduced restriction sites (Table S7) and inserted into pSU11, which was cut by the same combinations of restriction enzymes. The resulting plasmids were sequenced and transconjugated into *B. cenocepacia* H111 by triparental mating using *E. coli* pRK2013^73,74^ as helper strain. *B. cenocepacia* H111 cells containing the transcriptional fusions were selected on *Pseudomonas* Isolation Agar (Sigma-Aldrich, St. Louis, MO, USA) plates containing 10 µg/ml gentamycin. For the *cyo* promotor fusion two different size of the promotor were cloned and tested for expression. Both fusions showed no expression in any tested conditions. Primers used to construct the second *cyo* promotor fusion are as well listed in Table S7. All plasmid constructs were also transconjugated into two regulatory deletion mutant strains published previously (Δ*roxS*,* Δanr*_*1*_*-anr*_*2*_)^23^.

### Mutant construction

Insertional mutagenesis using the suicide plasmid pSHAFT2 was used to create the insertional *cio-1* mutant (*I35_RS29260*) as previously described^23,75,76^. In brief, a 282 bp fragment of the gene *I35_RS29260* was amplified by PCR using HF phusion polymerase (Sigma, St. Louis, MO, USA, F-530 L) and the following primer pairs: cio-1_in_Fw and cio-1_in_Rv. The plasmid and the amplified fragment were cut by *EcoRI HF* and thereafter ligated into pSHAFT2. The orientation of the fragment in the plasmid was checked by PCR and by Sanger sequencing. The plasmid harboring the fragment in forward was transconjugated into *B. cenocepacia* H111 wild-type via triparental mating.

### β-Galactosidase assay

β-galactosidase assay was performed according to the Miller method^69^. Generally, 1–5 ml cultures were harvested and resuspended in 200–600 µl of fresh Z-Buffer (containing 2-mercaptoethanol, Sigma-Aldrich, St. Louis, MO, USA). The OD_600_ was adjusted to 0.2–0.4 in 1 ml fresh Z buffer containing 25 µl 10% SDS and 25 µl chloroform (Sigma-Aldrich, St. Louis, MO, USA). After mixing the samples and incubation for 10 min at room temperature, 200 µl of ONPG (o-Nitrophenyl-β-D-galactopyranosid, 4 mg/ml) were added. The reaction was stopped by addition of 0.5 ml 1 M Na_2_CO_3_ after ca. 200 s and the samples were centrifuged for 10 min at maximum speed (13,200 rpm) and OD_420_ was measured.

### RT-qPCR analysis

The expression of *cio-1* (*I35_RS29260*) and *cio-2* (*I35_RS33335*) was analyzed with a Mx3000P instrument using Brilliant III Ultra-Fast SYBR^®^ Green QPCR Master Mix (Agilent, Switzerland). As template cDNA obtained from two biological replicates was prepared as previously described^21,77^. Each qPCR reaction contained 15 µl 2× Brilliant III Ultra-Fast SYBR^®^ Green QPCR Master Mix, 5 µM of individual primers and 15, 7.5 and 3.75 ng of cDNA with each dilution run in technical triplicates (total volume of 30 µl). Fold-changes in transcription were calculated as described previously^78^. Primers used are listed in Table S7.

### Characterization of the terminal oxidases

For characterization of the expression pattern of the terminal oxidases during aerobic growth, overnight cultures were washed with fresh medium and adjusted to a starting OD_600_ of 0.05 in 50 ml LB or ABC media in 250 ml flasks. Growth was measured for 24 h and β-galactosidase activity was assessed at different time points during the aerobic growth (mid exponential, late exponential, beginning stationary and late stationary phase) by withdrawing 2 ml of the growing culture. Expression differences of the terminal oxidases in the Δ*roxS* and *Δanr*_*1*_*-anr*_*2*_ mutant variants were measured from overnight cultures in 5 ml LB or ABC in 14 ml capped tubes. For micro-oxic growth, bacterial cultures were adjusted to a starting OD_600_ of 0.05 in 150 ml LB or ABC in 250 ml flaks and shaken at 200 rpm for 20 h. β-galactosidase activity of the terminal oxidases was measured after 20 h. In a test series, the β-galactosidase assay of the *cyd* construct was measured after growth in different medium volumes (25 ml, 50 ml, 75 ml, 150 ml) in a 250 ml flask to vary the oxygen tension as described by Cooper et al.^13^. The expression pattern of the different terminal oxidases was assessed on a solid surface by spreading the reporter fusion strains on LB or ABC plates (1.2%) and incubating them for 2 to 3 days at 37 °C. For cyanide experiments, overnight cultures were washed and adjusted in LB supplemented with 300 or 600 µM potassium cyanide to OD_600_ of 0.05 in a final volume of 5 ml in 14 ml capped tubes. The culture was grown to an OD_600_ between 0.3 and 0.6, pelleted and further assessed by the β-galactosidase assay. For the supernatant assay, *P. aeruginosa* PA14 wild-type and *P. aeruginosa* PA14 *hcnC* mutant strains^79^ were grown in 120 ml LB in a 250 ml flask overnight at 200 rpm at 37 °C. The *hcnC* deletion mutation was verified by PCR and absence of cyanide in the supernatant was tested with a filter detection assay^80^. The cultures were centrifuged (7 min, 5000 rpm) and the supernatant was filtered into 50 ml falcon tubes using a 0.2 μm filter. *B. cenocepacia* H111 wild-type and the *cio-1* insertional mutant strain were inoculated (OD_600_ 0.05 in 6 ml) in 14 ml tubes containing 50% LB and 50% supernatant of the respective *P. aeruginosa* strains. To assess if *B. cenocepacia* H111 produces cyanide a filter detection assay was performed with *B. cenocepacia* H111 culture either grown on solid agar plates (LB) or in LB liquid medium supplemented with 10 mM glycine (Cytiva, Buckinghamshire, UK). As positive control, *P. aeruginosa* was used and as negative control *E. coli*, which is unable to produce cyanide. The filter discs were prepared as follows: 50 mg copper(II) ethylacetoacetate (Sigma-Aldrich, St. Louis, MO, USA), and 50 mg 4,4-methylenebis(N, N-dimethylaniline) (Sigma Aldrich, St. Louis, MO, USA) were mixed into 10 ml chloroform and the filters were soaked in it for 10 s and dried overnight^80^.

## Electronic supplementary material

Below is the link to the electronic supplementary material.

Supplementary Material 1

## Data Availability

The dataset analyzed during the current study is available from the corresponding author on reasonable request.
