# Supplementary material for: Insights into the diverse roles of the terminal oxidases in Burkholderia cenocepacia H111
Source: Sci Rep. 2025 Jan 18;15:2390. doi: 10.1038/s41598-025-86211-8 (PMC11742914; doi:10.1038/s41598-025-86211-8)
Supplement: Supplementary file 1 — Supplementary Material 1 [file 41598_2025_86211_MOESM1_ESM.docx]

**Insights into the diverse roles of the terminal oxidases in *Burkholderia cenocepacia* H111**

**Sarah Paszti^1+^, Olivier Biner^1+*^, Yilei Liu, Kim Bolli^1^, Sarah Dorothy Jeggli^1^, Gabriella Pessi^1*^, Leo Eberl^1*^**

^1^ Department of Plant and Microbial Biology, University of Zurich, Zollikerstrasse 107, 8008 Zürich, Switzerland

^+^ Authors contributed equally

* Corresponding authors: [binerolivier@gmail.com](mailto:binerolivier@gmail.com), [gabriella.pessi@botinst.uzh.ch](mailto:gabriella.pessi@botinst.uzh.ch) and [leberl@botinst.uzh.ch](mailto:leberl@botinst.uzh.ch)

**Supplementary figures and tables**

**
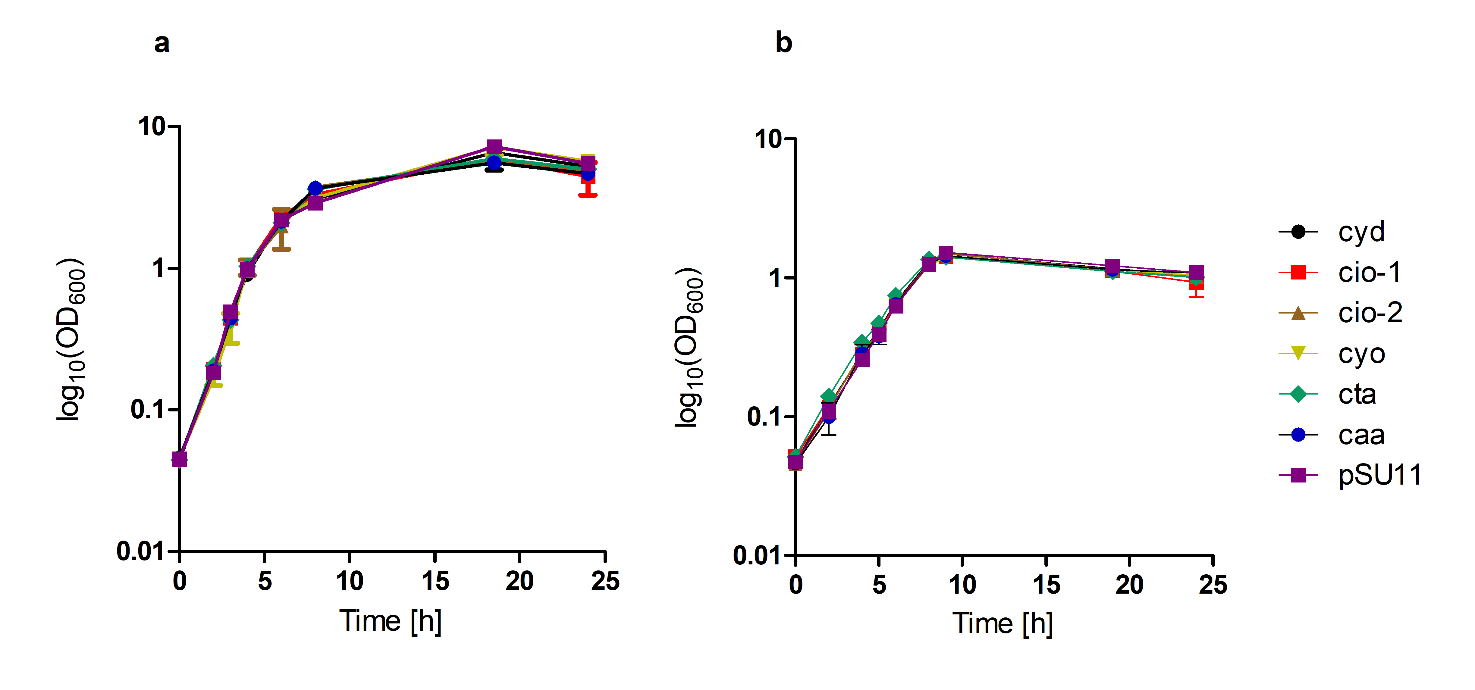
**

**Figure S1**: Growth of the B. cenocepacia H111 wild-type lacZ reporter strains. **a** Growth of the reporter strains in LB for 24 h at 200 rpm at 37 °C. **b** Growth of the reporter strains in ABC for 24 h at 200 rpm at 37 °C. Error bar = standard deviation (SD) where n=3. As a control the empty plasmid pSU11 was added into B. cenocepacia H111 (pSU11).


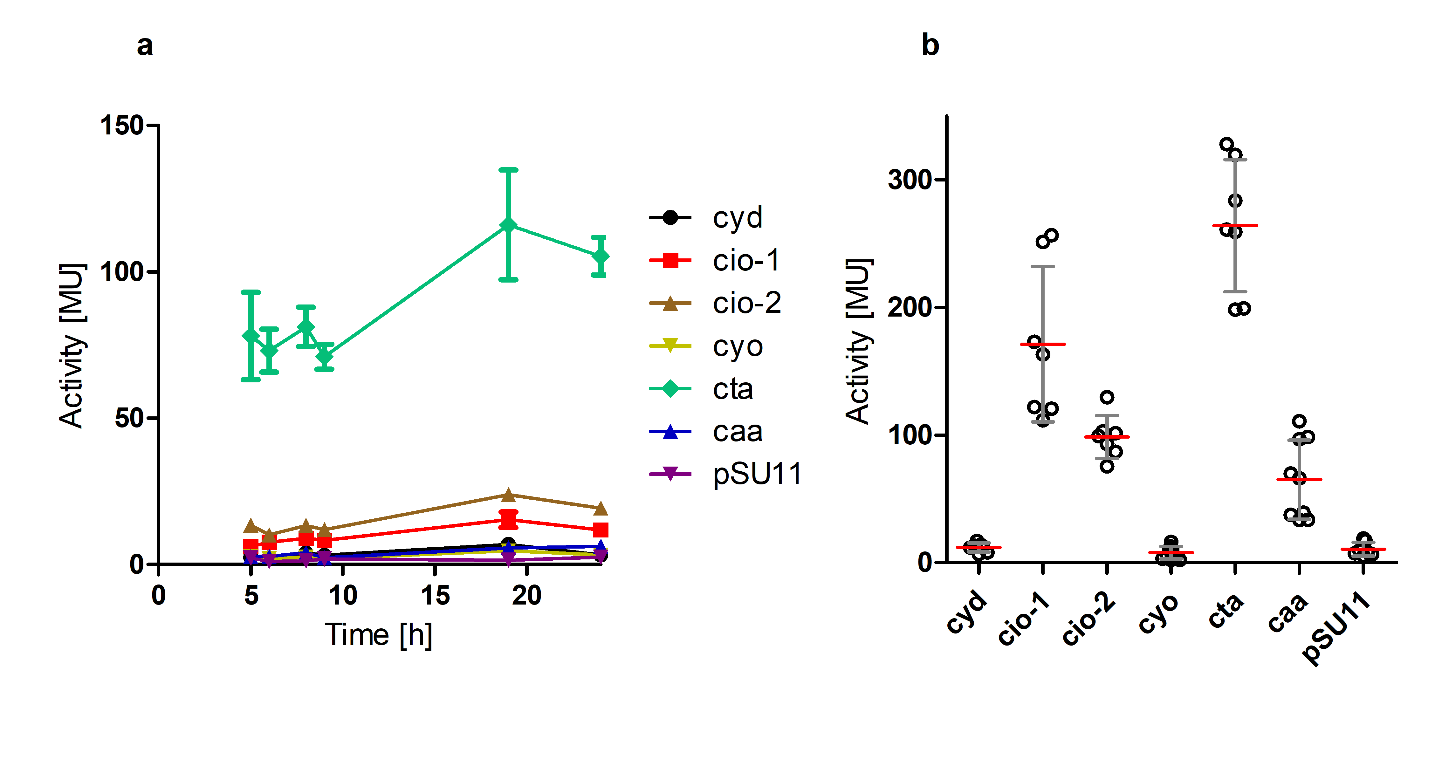


**Figure S2**: Expression pattern of the six terminal oxidases using lacZ reporter constructs. **a** Growth-dependent expression of the six terminal oxidases in liquid minimal medium (ABC). **b** Expression pattern of the terminal oxidases grown on solid ABC agar plates. MU = Miller units, Error bar = mean and SD where n=3 for **a** and n>3 for **b**. As a control the empty plasmid pSU11 was added into B. cenocepacia H111 (pSU11).

**Table S1:** Blast searches using blastP and blastX NCBI ^[1]^ . Given are coverage/identity in percentage. / = not performed.

|  | ***B. cenocepacia H111*** | ***E. coli K-12*** | ***P. aeruginosa PAO1*** | ***P. denitrificans PD1222*** | ***T. thermophilus***  ***HB8*** |
| --- | --- | --- | --- | --- | --- |
|  |  |  |  |  |  |
| ***cyd*** | *cydA* | 99/65 | / | / | / |
|  | *cydB* | 100/54 | / | / | / |
|  | *cydX* | 60/60 | / | / | / |
|  |  |  |  |  |  |
| ***cio-1*** | *cio1A* | / | 98/65 | / | / |
|  | *cio1B* | / | 99/59 | / | / |
|  | *cio1P* | / | / | / | / |
|  |  |  |  |  |  |
| ***cio-2*** | *cio2A* | / | 93/62 | / | / |
|  | *cio2B* | / | 100/56 | / | / |
|  |  |  |  |  |  |
| ***cyo*** | *cyoA* | / | 94/58 | / | / |
|  | *cyoB* | / | 97/69 | / | / |
|  | *cyoC* | / | 96/63 | / | / |
|  | *cyoD* | / | 89/52 | / | / |
|  |  |  |  |  |  |
| ***caa*** | *caaA* | / | / | / | 97/38 |
|  | *caaB* | / | / | / | 89/35 |
|  | *caaC* | / | / | / | / |
|  | *caaP* | / | / | / | / |
|  |  |  |  |  |  |
| ***cta*** | *ctaC* | / | / | 52/35 | / |
|  | *ctaD* | / | / | 98/53 | / |
|  | *ctaE* | / | / | 99/38 | / |
|  | *ctaG* | / | / | 83/36 | / |
|  | *ctaP1* | / | / | / | / |
|  | *ctaP2* | / | / | / | / |
|  | *ctaP3* | / | / | / | / |

**Table S2:** Expression (Miller unit) of B. cenocepacia H111 wild-type strains grown in LB liquid and on LB solid medium.

| Terminal oxidase | Liquid culture | | | | | | Plate |
| --- | --- | --- | --- | --- | --- | --- | --- |
|  | ***Mid exponential (3 h)***  ***OD_600_=0.5*** | ***Late exponential (4 h)***  ***OD_600_=1*** | ***Early stationary (6 h)***  ***OD_600_=3*** | ***Early stationary (8.0 h)***  ***OD_600_=3.8*** | ***Late stationary (18 h)***  ***OD_600_=7*** | ***Late stationary (24 h)***  ***OD_600_=5*** |  |
| *cyd* | 15±4 | 8±1 | 39±6 | 41±5 | 85±12 | 89±7 | 42±28 |
| *cio-1* | 12±1 | 16±1 | 34±5 | 32±5 | 73±15 | 88±24 | 843±166 |
| *cio-2* | 12±2 | 11±1 | 27±2 | 27±2 | 36±3 | 42±7 | 73±49 |
| *cyo* | 6±1 | 5±0 | 10±0 | 8±0 | 14±2 | 16±1 | 12±5 |
| *cta* | 116±5 | 119±5 | 140±15 | 127±9 | 135±5 | 155±8 | 148±62 |
| *caa* | 5±1 | 3±1 | 6±0 | 3±1 | 6±0 | 6±0 | 11±7 |
| control (pSU11) | 4±0 | 2±1 | 4±0 | 3±0 | 3±2 | 6±0 | 6±3 |

**Table S3:** Expression (Miller unit) of B. cenocepacia H111 wild-type strains grown in ABC liquid and on solid ABC medium.

| Terminal oxidase | Liquid culture | | | | | | Plate |
| --- | --- | --- | --- | --- | --- | --- | --- |
|  | ***Mid exponential (5 h)***  ***OD_600_=0.4*** | ***Late exponential (6 h)***  ***OD_600_=0.7*** | ***Early stationary***  ***(8 h)***  ***OD_600_=1.3*** | ***Mid stationary (9.0 h)***  ***OD_600_=1.5*** | ***Late stationary (19 h)***  ***OD_600_=1.15*** | ***Late stationary (24 h)***  ***OD_600_=1.0*** |  |
| *cyd* | 2±1 | 2±1 | 4±0 | 3±1 | 7±1 | 3±0 | 12±3 |
| *cio-1* | 6±1 | 8±0 | 9±1 | 8±1 | 15±2 | 12±1 | 171±56 |
| *cio-2* | 13±1 | 10±0 | 13±1 | 12±1 | 24±1 | 19±1 | 98±16 |
| *cyo* | 3±0 | 2±0 | 2±0 | 2±0 | 5±1 | 3±1 | 9±5 |
| *cta* | 78±12 | 73±6 | 81±5 | 71±3 | 116±15 | 105±5 | 264±48 |
| *caa* | 3±1 | 3±0 | 4±1 | 2±1 | 6±0 | 6±2 | 65±29 |
| control (pSU11) | 2±1 | 1±1 | 1±0 | 2±0 | 1±1 | 2±0 | 10±5 |

**Table S4:** Expression (Miller unit) of B. cenocepacia H111 wild-type strains grown at low oxygen in LB and ABC (150 ml in 250 ml flask) and in LB and LB supplemented with 600 µM KCN.

| Terminal oxidase | Low-oxygen experiment | | KCN experiment | |
| --- | --- | --- | --- | --- |
|  | **LB** | **ABC** | **LB** | **LB + 600 µM KCN** |
| *cyd* | 287±31 | 36±2 | 14±4 | 33±9 |
| *cio-1* | 109±10 | 20±3 | 19±3 | 837±95 |
| *cio-2* | 52±9 | 41±3 | 15±2 | 85±16 |
| *cyo* | 16±1 | 8±1 | 6±1 | 18±5 |
| *cta* | 149±7 | 129±27 | 142±21 | 230±36 |
| *caa* | 2±1 | 11±1 | 4±1 | 4±1 |
| control (pSU11) | 3±1 | 5±1 | 3±2 | 5±1 |

**Table S5:** Expression (Miller unit) of B. cenocepacia H111 wild-type, ∆roxS and ∆anr_1_anr_2_ grown in ABC liquid and on ABC solid medium.

| Terminal oxidase | Liquid culture | | | Plate | | |
| --- | --- | --- | --- | --- | --- | --- |
|  | **Wild-type** | **∆*roxS*** | **∆*anr_1_anr_2_*** | **Wild-type** | **∆*roxS*** | **∆*anr_1_anr_2_*** |
| *cyd* | 7±4 | 5±2 | 5±2 | 12±3 | 6±2 | 4±2 |
| *cio-1* | 25±7 | 3±1 | 379±140 | 171±56 | 8±5 | 524±207 |
| *cio-2* | 33±16 | 33±10 | 31±0 | 98±16 | 72±15 | 78±17 |
| *cyo* | 8±8 | 3±2 | 5±0 | 9±5 | 7±4 | 7±3 |
| *cta* | 126±46 | 119±10 | 156±7 | 264±48 | 220±9 | 198±33 |
| *caa* | 7±4 | 6±2 | 10±4 | 65±29 | 59±23 | 26±19 |
| control (pSU11) | 6±6 | 1±1 | 5±1 | 10±5 | 5±1 | 4±2 |

**Table S6:** qPCR results for cio-1 (I35_RS29260) and cio-2 (I35_RS33335) in the following conditions C = cyanide, E = exponential phase and P = plate shown as fold change (FC) of transcript expression.

| **Gene** | **Description** |  | **FC C vs E** | **FC P vs E** |
| --- | --- | --- | --- | --- |
| ***cio_1*** | Cyanide insensitive terminal oxidase 1 |  | 66 ± 6.1 | 4.2 ± 0.5 |
| ***cio_2*** | Cyanide insensitive terminal oxidase 2 |  | 43 ± 5.6 | 8.3 ± 0.2 |

**Table S7**: List of strains, oligonucleotides and plasmids used in this study.

| **Strain or plasmid** | **Description** | **Reference** |
| --- | --- | --- |
| Strains |  |  |
| *E. coli* |  |  |
| cc118λ-pir | Δ(ara-leu) *araD* Δ*lacX74 galE galK phoA20 thi1 rpsE rpoB argE(Am) recAl* λ pir; Strep^R^ | ^[2]^ |
| *P. aeruginosa* |  |  |
| UCBPP-PA14 | Wild-type, clinical isolate from a burn wound | ^[3]^ |
| *hcnC* | MAR2xT7::*PA14_36310* mutant of PA14 wild-type (*hcnC*), Gm^R^ | ^[4]^ |
| *B. cenocepacia* |  |  |
| wild-type | CF isolate from Germany, genomovar III | ^[5,6]^ |
| Δ*roxS* | Mutant in the two-component family of histidine kinase Δ*roxS (I35_RS15825)* | ^[7]^ |
| ∆*anr_1_-anr_2_* | Δ*anr_1_ (I35_RS23120*) deletion and *anr_2_ (*pSHAFT2*::I35_RS16810)* double mutant, Km^R^ , Cm^R^ | ^[7]^ |
| *cyd* | H111 P*cyd*::*lacZ* transcriptional fusion, Gm^R^ | This study |
| *cio-1* | H111 P*cio*-1::*lacZ* transcriptional fusion, Gm^R^ | This study |
| *cio-2* | H111 P*cio*-2::*lacZ* transcriptional fusion, Gm^R^ | This study |
| *cyo* | H111 P*cyo*::*lacZ* transcriptional fusion, Gm^R^ | This study |
| *cyo 2* | H111 P*cyo*::*lacZ* transcriptional fusion, Gm^R^ | This study |
| *cta* | H111 P*cta*::*lacZ* transcriptional fusion, Gm^R^ | This study |
| *caa* | H111 P*caa*::*lacZ* transcriptional fusion, Gm^R^ | This study |
| pSU11 | H111 Pempty::*lacZ* transcriptional fusion, Gm^R^ | This study |
| Δ*roxS*-P*cyd*-*lacZ* | Deletion mutant Δ*roxS* harbouring P*cyd*::*lacZ* transcriptional fusion, Gm^R^ | This study |
| Δ*roxS*-P*cio*-1-*lacZ* | Deletion mutant Δ*roxS* harbouring P*cio*-1::*lacZ* transcriptional fusion, Gm^R^ | This study |
| Δ*roxS*-P*cio*-2-*lacZ* | Deletion mutant Δ*roxS* harbouring P*cio*-2::*lacZ* transcriptional fusion, Gm^R^ | This study |
| Δ*roxS*-P*cyo*-*lacZ* | Deletion mutant Δ*roxS* harbouring P*cyo*::*lacZ* transcriptional fusion, Gm^R^ | This study |
| Δ*roxS*-P*cta*-*lacZ* | Deletion mutant Δ*roxS* harbouring P*cta*::*lacZ* transcriptional fusion, Gm^R^ | This study |
| Δ*roxS*-P*caa*-*lacZ* | Deletion mutant Δ*roxS* harbouring P*caa*::*lacZ* transcriptional fusion, Gm^R^ | This study |
| Δ*roxS*-Pempty-*lacZ* | Deletion mutant Δ*roxS* harbouring Pempty::*lacZ* transcriptional fusion, Gm^R^ | This study |
| Δ*anr_1_anr_2_*-P*cyd*-*lacZ* | Double mutant Δ*anr_1_anr_2_* harbouring P*cyd*::*lacZ* transcriptional fusion, Gm^R^, Km^R^, Cm^R^ | This study |
| Δ*anr_1_anr_2_*-P*cio*-1-*lacZ* | Double mutant Δ*anr_1_anr_2_* harbouring P*cio*-1::*lacZ* transcriptional fusion, Gm^R^, Km^R^, Cm^R^ | This study |
| Δ*anr_1_anr_2_*-P*cio*-2-*lacZ* | Double mutant Δ*anr_1_anr_2_* harbouring P*cio*-2::*lacZ* transcriptional fusion, Gm^R^, Km^R^, Cm^R^ | This study |
| Δ*anr_1_anr_2_*-P*cyo*-*lacZ* | Double mutant Δ*anr_1_anr_2_* harbouring P*cyo*::*lacZ* transcriptional fusion, Gm^R^, Km^R^, Cm^R^ | This study |
| Δ*anr_1_anr_2_*-P*cta*-*lacZ* | Double mutant Δ*anr_1_anr_2_* harbouring P*cta*::*lacZ* transcriptional fusion, Gm^R^, Km^R^, Cm^R^ | This study |
| Δ*anr_1_anr_2_*-P*caa*-*lacZ* | harbouring | This study |
| Δ*anr_1_anr_2_*-Pempty-*lacZ* | Double mutant Δ*anr_1_anr_2_* harbouring Pempty::*lacZ* transcriptional fusion, Gm^R^, Km^R^, Cm^R^ | This study |
| *cio-1*_in_mutant | Insertional mutant in *cio-1* (*I35_RS29260*), Cm^R^ | This study |
| Plasmids |  |  |
| pRK2013 | Helper plasmid; Km^R^ | ^[8]^ |
| pSHAFT2 | Broad-host-range suicide plasmid, mobilisable for conjugation; Cm^R^ | ^[9]^ |
| pSU11 | *lacZ* reporter plasmid, Gm^R^ | ^[10]^ |
| P*cyd*-*lacZ* | *lacZ* reporter plasmid containing the promotor region (389 pb) of the *cyd* cluster, Gm^R^ | This study |
| P*cio*-1-*lacZ* | *lacZ* reporter plasmid containing the promotor region (508 pb) of the *cio-1* cluster, Gm^R^ | This study |
| P*cio*-2-*lacZ* | *lacZ* reporter plasmid containing the promotor region (465 pb) of the *cio-2* cluster, Gm^R^ | This study |
| P*cyo*-*lacZ_1* | *lacZ* reporter plasmid containing the promotor region (318 pb) of the *cyo* cluster, Gm^R^ | This study |
| P*cyo*-*lacZ_2* | *lacZ* reporter plasmid containing the promotor region (328 pb) of the *cyo* cluster, Gm^R^ | This study |
| P*cta*-*lacZ* | *lacZ* reporter plasmid containing the promotor region (207 pb) of the *cta* cluster, Gm^R^ | This study |
| P*caa*-*lacZ* | *lacZ* reporter plasmid containing the promotor region (302 pb) of the *caa* cluster, Gm^R^ | This study |
| pSHAFT2_cio-1 | pSHAFT harbouring a 282 bp fragment of *cio-1* (*I35_RS29260*), Cm^R^ | This study |
| Oligonucleotides |  |  |
| Pcyd_for | gcgcctcgagGGCAATTTAGAGCGCTTCGT | This study |
| Pcyd_rev | gcgcaagcttGCGAAGGCTCTTTATTGATCGA | This study |
| Pcio-1_for | gcgcctcgagCGCGAGCTTCAATCCATTCA | This study |
| Pcio-1_rev | gcgcaagcttCGAAAGTGCGGTGTCCATAC | This study |
| Pcio-2_for | gcgcgtcgacCGGGTGCAGGACTCCATG | This study |
| Pcio-2_rev | gcgcaagcttCAATCGGGCGAGATGGAAC | This study |
| Pcyo_for_1 | gcgcctcgagGACGCACGACTGATCCCG | This study |
| Pcyo_rev_1 | gcgcaagcttGCCGACCAGCCTCTTGAA | This study |
| Pcyo_for_2 | tccctcgagCGAAATGACCGGTTGACATC | This study |
| Pcyo_rev_2 | tccaagcttATGCATGTCAACAGGGCCTT | This study |
| Pcta_for | gcgcctcgagTCGGTAAAGCGGTAAAGAGG | This study |
| Pcta_rev | gcgcgtcgacTGCATCGGACTCGTCGCC | This study |
| Pcaa_for | gcgcctcgagGTCTTCACGGTCGAGTCCTC | This study |
| Pcaa_rev | gcgcgtcgacAACGAGACTCCTTGCCGG | This study |
| pSHAFT_Fw | CTTCAGCTGATGTGTGATAACATACT | ^[11]^ |
| cio-1_in_Fw | tccgaattcTGCCTGTTCTGGTCGAAGAT | This study |
| cio-1_in_Rv | tccgaattcGAGGATCCAGAACGTCGAGA | This study |
| pSU11_lacZ_rev | TGCTGCAAGGCGATTAAG | This study |
| pSU11_seq2_for | TTACAAGCATAAAGCTGACTCTAG | This study |
| qPCR_cio_1a_F | ATGAATCGAAGGGGTTCCAG | This study |
| qPCR_cio_1a_R | ACGAACGTCATCATCGACAG | This study |
| qPCR_cio_2a_F | AAGTCCTGACGGCGTTCTT | This study |
| qPCR_cio_2a_R | AGGCCGTTCTCGATCTTGTA | This study |

**References**

1. Agarwala, R. *et al.* Database resources of the national center for biotechnology information. *Nucleic Acids Res.* **46**, D8–D13 (2018).

2. Herrero, M., De Lorenzo, V. & Timmis, K. N. Transposon vectors containing non-antibiotic resistance selection markers for cloning and stable chromosomal insertion of foreign genes in gram-negative bacteria. *J. Bacteriol.* **172**, 6557–6567 (1990).

3. Rahme, L. G. *et al.* Common virulence factors for bacterial pathogenicity in plants and animals. *Science (80-. ).* **268**, 1899–1902 (1995).

4. Liberati, N. T. *et al.* An ordered, nonredundant library of *Pseudomonas aeruginosa* strain PA14 transposon insertion mutants. *Proc. Natl. Acad. Sci. U. S. A.* **103**, 2833–2838 (2006).

5. Romling, U., Wingender, J., Muller, H. & Tummler, B. A major *Pseudomonas aeruginosa* clone common to patients and aquatic habitats. *Appl Env. Microbiol* **60**, 1734–1738 (1994).

6. Gotschlich, A. *et al.* Synthesis of multiple N-acylhomoserine lactones is wide-spread among the members of the *Burkholderia cepacia* complex. *Syst Appl Microbiol* **24**, 1–14 (2001).

7. Paszti, S. *et al.* Identification of key factors for anoxic survival of *B. cenocepacia* H111. *Int. J. Mol. Sci.* **23**, 4560 (2022).

8. Phadnis, S. H. & Berg, D. E. Identification of base pairs in the outside end of insertion sequence *IS50* that are needed for *IS50* and Tn*5* transposition. *Proc Natl Acad Sci U S A* **84**, 9118–9122 (1987).

9. Shastri, S. *et al.* An efficient system for the generation of marked genetic mutants in members of the genus *Burkholderia*. *Plasmid* **89**, 49–56 (2017).

10. Regulation, Q. *et al.* A *Burkholderia cenocepacia* orphan LuxR homolog is involved in quorum-sensing regulation. *J. Bacteriol.* **191**, 2447–2460 (2009).

11. Lardi, M., Liu, Y., Purtschert, G., de Campos, S. B. & Pessi, G. Transcriptome analysis of *Paraburkholderia phymatum* under nitrogen starvation and during symbiosis with *Phaseolus vulgaris*. *Genes (Basel).* **8**, 389 (2017).
